# Constitutive Optimized Production of Streptokinase in *Saccharomyces cerevisiae* Utilizing Glyceraldehyde 3-Phosphate Dehydrogenase Promoter of *Pichia pastoris*

**DOI:** 10.1155/2013/268249

**Published:** 2013-09-22

**Authors:** Ravi N. Vellanki, Ravichandra Potumarthi, Kiran K. Doddapaneni, Naveen Anubrolu, Lakshmi N. Mangamoori

**Affiliations:** ^1^Centre for Biotechnology, Institute of Science and Technology, Jawaharlal Nehru Technological University, Kukatpally, Hyderabad, Andhra Pradesh 500085, India; ^2^Ontario Cancer Institute, Princess Margaret Cancer Centre, 610 University Avenue, Toronto, ON, Canada M5G 2M9; ^3^Department of Chemical Engineering, Monash University, Clayton, VIC 3800, Australia

## Abstract

A novel expression vector constructed from genes of *Pichia pastoris* was applied for heterologous gene expression in *Saccharomyces cerevisiae*. Recombinant streptokinase (SK) was synthesized by cloning the region encoding mature SK under the control of glyceraldehyde 3-phosphate dehydrogenase (*GAP*) promoter of *Pichia pastoris* in *Saccharomyces cerevisiae*. SK was intracellularly expressed constitutively, as evidenced by lyticase-nitroanilide and caseinolytic assays. The functional activity was confirmed by plasminogen activation assay and *in vitro* clot lysis assay. Stability and absence of toxicity to the host with the recombinant expression vector as evidenced by southern analysis and growth profile indicate the application of this expression system for large-scale production of SK. Two-stage statistical approach, Plackett-Burman (PB) design and response surface methodology (RSM) was used for SK production medium optimization. In the first stage, carbon and organic nitrogen sources were qualitatively screened by PB design and in the second stage there was quantitative optimization of four process variables, yeast extract, dextrose, pH, and temperature, by RSM. PB design resulted in dextrose and peptone as best carbon and nitrogen sources for SK production. RSM method, proved as an efficient technique for optimizing process conditions which resulted in 110% increase in SK production, 2352 IU/mL, than for unoptimized conditions.

## 1. Introduction

Streptokinase (SK) is a nonenzymatic thrombolytic protein secreted by Lancefield group C strains of beta hemolytic streptococci and is important in their virulence [1, 2]. A growing thrombotic mass may lead to partial or complete thrombotic arterial occlusion and end-organ ischemia or infarction. SK activates the fibrinolytic system indirectly by forming a 1 : 1 stoichiometric complex with plasminogen or plasmin. When fibrin thrombi develop, plasminogen adsorbs to the clot and SK penetrates the clot, activating plasminogen to plasmin, a proteolytic enzyme which dissolves the clot from within. SK has been used successfully in the treatment of pulmonary embolism, myocardial infarction, arterial/deep vein thrombosis, and clotted arteriovenous fistulae [1, 3].

Fibrinolytic molecules (enzymes/nonenzymes) were extracted, purified to homogeneity from a variety of microbial sources, and tested for their efficacy and toxigenicity [1, 4–6]. The *Streptococcus equisimilis *H46A strain expressing SK is pathogenic and also secretes other potent antigenic toxins. The *skc *gene encoding SK (47 kDa) is produced in a heterologous host that is generally regarded as safe (GRAS) organism. A variety of bacterial and yeast hosts were successfully exploited for the production of active SK [7, 8]. *S. cerevisiae*, due to its extensive data on gene manipulation tools, is the traditional host for the production of heterologous proteins. The advent of alcohol oxidase (*AOX*) expression system of *P. pastoris* led to successful protein industrialization. The disadvantages associated with *AOX* expression system are chiefly that the methanol utilization led to the development of alternate promoter systems *GAP*, *FLD,* and *ICL1* for protein expression. Of the above promoters, *GAP-*(glyceraldehyde 3 phosphate dehydrogenase-) based promoter system has been extensively applied for constitutive continuous bioprocess [7, 9–12]. SK was expressed as intracellular/secretory molecule in *P. pastoris* and *Schizosaccharomyces pombe* [7, 13–15].

Microtiter analyses have been used to determine protein contents, enzyme activities, ligand binding and are increasingly popular with the use of colorimetry/ fluorescence for efficiency and throughput. They have also been used to determine growth and lysis by spheroplast lysis assay [16–18]. We describe here the adaptation of this assay in conjunction with a chromogenic substrate for qualitative detection of SK levels in yeast clones [19].

SK production was maximized by screening carbon and nitrogen sources using two-evel Plackett-Burman (PB) design [20]. Conventionally, optimization of the process variables involves changing one variable at a time while others are held constant. Practically, this method is laborious to test every possible combination of test variables as it results in large number of experiments [21, 22]. Besides, it does not consider the effect of interactions of various parameters. Alternatively, response surface methodology (RSM) can be used to evaluate and understand interaction among process variables [23]. RSM was successfully applied into bioprocess parameter optimization [24–28].

We have previously reported the production of hepatitis B surface antigen (HBsAg) in *S. cerevisiae* utilizing *GAP* promoter of *P. pastoris* [12]. The same vector backbone was used for SK expression. In the current, investigation we have explored the following: (1) intracellular expression of SK in *S. cerevisiae* utilizing *GAP* promoter of *P. pastoris* (2) optimization of nutrients for SK production using the above expression system by response surface methodology (RSM) and comparison between normal and baffled flasks, and (3) qualitative SK detection by Lyticase-nitroanilide assay (LNA). We report the successful constitutive intracellular expression of SK in *S. cerevisiae*, detection by LNA, followed by increased production using baffled-flask design after RSM. Significantly, peptone and dextrose have shown the maximal SK production. We have obtained a level of expression considerable to that achieved in other yeast systems.

## 2. Materials and Methods

### 2.1. Materials

Media for bacterial and yeast growth Todd-Hewitt broth himedia (Mumbai, India) and yeast extract peptone dextrose (YPD) from USB (Cleveland, OH) were used. Restriction enzymes, T4 DNA ligases, were purchased from New England Biolabs (Beverly, MA). PCR was performed with Eppendorf mastercycler using *Pfu *DNA polymerase (Stratagene, CA, USA). Plasmid transformation and retainment were done in *Escherichia coli *DH5*α* from Gibco BRL (Gaithersburg, MD). *Saccharomyces cerevisiae INVSC1 *(*MATa, his3D1, leu2, trp1-289,* and *ura3-52*) from Invitrogen (Carlsband, CA, USA) is used in expression studies. Human plasminogen, thrombin, and fibrinogen were purchased from Calbiochem (La Jolla, CA)/Sigma (St.Louis, MO). Nitroanilide substrate S-2251 is purchased from Fluka (Buchs/Switzerland).

### 2.2. Growth Media and Conditions

All *E. coli *DH5 *α* based experiments were performed in low-salt luria broth (1% tryptone, and 0.5% yeast extract, 0.5% NaCl pH 7.5) medium with Zeocin (25 *μ*g/mL). Transformants of *S. cerevisiae* were selected on YPD (1% yeast extract, 2% peptone, and 2% dextrose) medium with Zeocin (200 *μ*g/mL) at 30°C. For protein expression, *S. cerevisiae* were cultured in YPD medium at 30°C.

### 2.3. Construction of Recombinant Streptokinase Expression Vector

**Figure 1:**
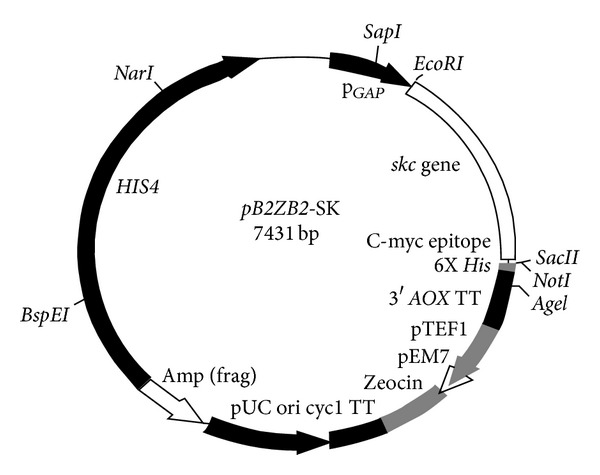
Plasmid map of expression vector *pB2ZB2*-SK constructed to express streptokinase in *S. cerevisiae*. 1.25 Kbp *skc *gene is operably cloned downstream to *GAP* promoter generating a recombinant *pB2ZB2*-SK expression vector. Appropriate restriction endonuclease sites are designated.

SK coding region was amplified by PCR using P1-CAGCAG**GAATTC**ATTGCTG GACCTGAGTGG, and P2-TCCC**CTCGAG**TTATTTGTCGTTAGGGTTATC primers from p*GAPZA*-SK vector [7] in a gene Eppendorf mastercycler PCR system and ligated to *pB2ZB2* vector [12] downstream of *GAP* promoter at *EcoRI* and *XhoI* sites. In-frame cloning of *skc* gene between *GAP* promoter and *AOX* TT terminator in the expression plasmid was analyzed by restriction digestion and DNA sequencing as described previously [7]. The resulting recombinant plasmid *pB2ZB2-*SK (Figure 1) was maintained in *E. coli* DH5 *α* with Zeocin as a selection pressure. *pB2ZB2-*SK was used as an integrative expression vector to transform *S. cerevisiae*.

### 2.4. Transformation and Screening of *S. cerevisiae* with pB2ZB2-SK

*S. cerevisiae* were transformed with *NarI*-linearized *pB2ZB2-*SK vector (7431 bp) by lithium acetate method and selected as detailed earlier [12]. The transformants were screened for P_*GAP*_-*skc*-_*AOX*_TT cassette by colony PCR [7].

#### 2.4.1. Lyticase Nitroanilide Assay (LNA Assay)

A part of the transformant colony was inoculated in a 96-well conical-bottomed microtest plate (Thermo Scientific Microtiter plates) with each well containing 300 *μ*L of YPD-Zeocin medium. The plate was properly sealed and incubated at 30°C, 100 rpm for 16 h to obtain exponential-phase cells. Doubling time of log phase for *Saccharomyces* in YPD medium is ~90 min. The density of cells in liquid culture was determined at A_600_ (Thermo Multiskan Spectrum spectrophotometer). A fresh 96-well conical-bottomed plate was inoculated with 0.3 OD cells with each well containing 300 *μ*L of YPD medium and incubated at 30°C, 100 rpm for 24 h. After incubation, cell density was determined and the plates were centrifuged for 5 min at 430 g (Eppendorf microcentrifuge 5430 R). The supernatant was discarded from each well using a vacuum pump. The cells were washed in PBS and centrifuged for 2 min at 430 g. The culture was diluted in Z buffer (60 mM Na_2_HPO_4_, 40 mM NaH_2_PO_4_, 10 mM KCl, 1 mM MgSO_4_, and 50 mM *β*-ME, pH 7.0) to obtain 0.6 OD. 50 *μ*L of lyticase (10 *μ*g) was added to the cell suspension and incubated for 30 min at 22°C with no shaking. This is followed by addition and incubation of 100 *μ*L of 0.01% Triton X-100 solution for 15 min at 22°C. After incubation, plates were centrifuged for 5–10 min at 430 g. If cell lysis is incomplete, freeze thawing at −80°C was done.

A 20 *μ*L of cell-lysis supernatant was transferred to flat-bottom 96-well microtitration plates (Thermo Scientific) and proceeded with S-2251 assay. Control samples were prepared by adding 20 *μ*L 0.1 M Tris-HCl buffer, pH 7.4, 30 *μ*L substrate S-2251 (0.6 mM), and 100 *μ*L plasminogen solution (0.1 CU/mL). In the test samples, the reaction was performed after the addition of 20 *μ*L SK, 30 *μ*L S-2251, and 100 *μ*L plasminogen solutions. The plate was immediately placed in the plate reader previously heated at 37°C. The absorbance of the wells was measured at 405 nm every 30 s for 30 min. Plates were shaken for 5 s before reading the absorbance using a Thermo Multiskan Spectrum spectrophotometer and analyzing with SkanIt software. A calibration curve was constructed from five measurements with reference standards (50–2500 IU/mL). Each SK dilution was assayed a minimum of three times. One unit of SK is defined as the amount of enzyme activity that converts 1 *μ*mol of substrate per min per Liter.

### 2.5. Time Course/Expression Experiments

*S. cerevisiae* clone with maximum SK expression (*S.cerv*-SK), screened by LNA assay, was cultured in a 10 mL YPD medium at 30°C for 24 hrs, 180 rpm. 1.0 OD of 24 hr culture was inoculated in a 25 mL YPD medium in a 150 mL normal/baffled flask [29]. A time course study was performed at 30°C, 180 rpm for 120 hrs to evaluate the growth and expression profile. *S. cerevisiae* harboring only the parent plasmid and untransformed strains were used as controls. Cell lysates of* S.cerv*-SK clone were prepared as discussed previously [12] and soluble SK was screened using chromogenic assay and clot lysis assay. The cell lysate was analyzed for expression of rSK or stored at −70°C.

### 2.6. Southern Analysis

Genomic DNA was isolated from *S. cerevisiae *culture as per the standard procedure [30]. Quantified genomic DNA (10 *μ*g) was restricted with *SapI* and *AgeI* and electrophoresed on 0.8% agarose gel. Resolved fragments were transferred to Hybond nylon membrane, probed with 1.63 Kbp P_*GAP*_-*skc*-_*AOX*_TT cassette fragment, and radiolabeled by nick translation kit (GIBCO-BRL) with [*α*-32P] dCTP. A P_*GAP*_-*skc*-_*AOX*_TT cassette fragment was generated by digestion of *pB2ZB2-*SK vector with *SapI* and *AgeI*. DNA from untransformed host was also included in the analysis. After hybridization and washing, P_*GAP*_-*skc*-_*AOX*_TT insertion to yeast genomic DNA was visualized by autoradiography. 

### 2.7. Activity Assay Methods

Plasminogen activation assay, fibrin plate lysis assay, and *in vitro* clot lysis assay were performed as described previously [19, 31–35]. Purified native SK (Streptase) was used as a standard. 

### 2.8. Carbon and Organic Nitrogen Sources Screening by Plackett-Burman Design

The aim of the present study was to screen significant carbon and organic nitrogen sources with respect to their main effects on SK production by PB design, but it is not intended to study interaction effects between various medium constituents. Different carbon sources, namely, dextrose, galactose, fructose, maltose, sucrose, lactose, and glycerol, and nitrogen sources, namely, yeast extract, tryptone, peptone, casamino acids, beef extract corn steep liquor, and polypeptone, were screened by PB design. Seven carbon and nitrogen sources were selected with each variable at two levels, high concentrations (+1; 2%) and low concentrations (−1; 1%), respectively, and tested for SK production. 

Experiments were carried in 250 mL Erlenmeyer flasks with 100 mL of production medium. Different carbon and nitrogen sources were screened at 1% yeast extract, 2% peptone, pH 7.0, 200 rpm, and 2% dextrose, pH 7.0, 200 rpm for SK production, respectively. Supplementary Tables S1 and S2, available online at http://dx.doi.org/10.1155/2013/268249, shows design matrix (eight experiments) generated by PB to screen carbon and nitrogen sources. Number of positive signs and negative signs per trial are (*k* + 1)/2 and (*k* − 1)/2, respectively. Here, *k* represents the number of variables, that is, 7 for both carbon and nitrogen sources. Columns of design matrix (supplementary Tables S1 and S2) should have equal number of positive and negative signs, meaning that each row represents a trial run and each column represents an independent variable. The effect of each variable was determined by the following equation:

(1) $$\begin{array}{c} E\left(x_{i}\right)=\;\frac{2\left(\sum {M_{i}}^{+}+{M_{i}}^{-}\right)}{N}, \end{array}$$

where *E*(*x*_*i*_) is the concentration effect of the tested variable; *M*_*i*_^+^ and *M*_*i*_^−^ are from the trials where the variable (*x*_*i*_) measured at high and low concentrations, respectively; *N* is the number of trials. STATISTICA 6.0 (Stat Soft, Inc, Tulsa, OK) software was used for regression and graphical analysis of the data obtained.

### 2.9. Experimental Design and Optimization of SK Production Medium by RSM [21–23]

Optimum SK production medium composition was achieved by estimating the levels of parameters using RSM. RSM is an empirical technique used for the evaluation of relationships between a cluster of controlled experimental factors and measured responses. A central composite design (CCD) was used for RSM studies. CCD has the total number of combinations 2^*k*^ + 2∗*k* + *n*_0_, where *k* is the number of independent variables and *n*_0_ is the number of repetitions of the experiments at the center point. Four important SK production medium components were selected by the best results of conventional (one at a time) approach. Further, these four parameters were evaluated for their interactive behaviors by using a statistical approach. The levels of four variables, namely, yeast extract, 2.6% (*x*_1_); dextrose, 2.7% (*x*_2_); pH, 7.1 (*x*_3_), and temperature, 30°C (*x*_4_) were coded at five levels −2, −1, 0, 1, and, 2 by using (2).

For statistical calculations, the variables *X*_*i*_ were coded as *x*_*i*_ according to the following transformation.

The range and levels of the variables in coded units for RSM studies are given in supplementary Table S3. Consider

(2) $$\begin{array}{c} x_{i}=\frac{X_{i}-X_{0}}{\Delta X}, \end{array}$$

where *x*_*i*_ is the dimensionless coded value of the variable *X*_*i*_, *X*_0_ is the value of the *X*_*i*_ at the center point, and Δ*X* is the step change.

The behavior of the system was explained by the following quadratic model (3).

(3) $$\begin{array}{c} Y=\beta_{0}+\sum \beta_{i}*x_{i}+\sum \beta_{ii}*x_{i}^{2}+\sum \beta_{ij}*x_{ij}, \end{array}$$

where *Y* is the predicted response, *β*_0_ is the intercept term, *β*_*i*_ is the linear effect, *β*_*ii*_ is the multiple effect, and *β*_*ij*_ is the interaction effect. The full quadratic equation for four factors is given by model (4).

(4) Y=β0+β1x1+β2x2+β4x3+β4x4 +β11x1∗x1+β12x1∗x2+β13x1∗x3 +β14x1∗x4+β22x2∗x2+β23x2∗x3 +β24x2∗x4+β33x3∗x3+β34x3∗x4 +β44x4∗x4.

In the present study, a 2^4^ full factorial central composite design with eight points and six replicates at central point was used to fit the second-order polynomial equation. This approach has resulted in 30 experiments. Regression and graphical analysis of data were carried out by STATISTICA 6.0 (Stat Soft, Inc, Tulsa, OK). Selection of optimum combination of four test variables for the better SK production was performed according to the CCD experimental plan (supplementary Table S4). Analysis of variance (ANOVA) was used for regression equations to obtain optimal levels of SK production as a function of the initial values of yeast extract, dextrose, pH, and temperature.

## 3. Results and Discussion

Streptokinase was expressed intracellularly with no posttranslational proteolysis in *P. pastoris *by *AOX1* [13] and *GAP* [7] promoters. Constitutive intracellular expression, continuous bioprocess in YPD medium for SK were achieved with *P. pastoris *[7]. Expression of SK in *S. cerevisiae* either by intracellular or secretory mode was not reported previously. The present study demonstrates the constitutive expression of rSK intracellularly in *S. cerevisiae* by employing the *GAP* promoter of *P. pastoris*. This is the first report of a *GAP* promoter operable, dominant selective, optimized constitutive intracellular expression of recombinant SK in *S. cerevisiae*.

### 3.1. Construction of Recombinant Expression Vectors

The *pB2ZB2* plasmid contains the P_*GAP*_-*skc*-_*AOX*_TT cassette, wild-type histidinol dehydrogenase (*HIS4*) gene for linearized integration into host genome and Zeocin cassette bi-functional in both *S. cerevisiae *and *E. coli* for the selection of transformants.The *skc* gene was cloned into the *pB2ZB2* vector generating recombinant expression vector *pB2ZB2-*SK (Figure 1). The cloning of *skc* gene downstream of *GAP* promoter was confirmed by PCR and nucleotide sequencing. Genetic markers like *HIS4* and *ARG4* of *S. cerevisiae* were used in episomal and integrative vectors in *P. pastoris *[36]. *SUC2* marker of *S. cerevisiae* was used as dominant selection in *P. pastoris *[36]. Constitutive promoters like *PGK1* (Phospho Glycerate Kinase) and *TDH3* (Triose Phosphate Dehydrogenase 3) of *S. cerevisiae* have been used for heterologous protein expression [36, 37].

*HIS4* locus encoding histidinol dehydrogenase in *P*. *pastoris* and *S. cerevisiae* shares more than 50% homology at DNA level. *NarI* restriction site in *HIS4* locus was chosen to linearize the recombinant *pB2ZB2*-SK vector, as the sequence homology around this site is more than 85%. Efficient integration to *S. cerevisiae HIS4* locus by homologous recombination with *P. pastoris HIS4* locus was successfully achieved. Integration of *skc* gene expression cassette was confirmed by PCR with promoter- and terminator-specific primers and by southern hybridization indicating that the expression of SK is in fact mediated by *GAP* promoter.

### 3.2. Generation of Constitutive *Saccharomyces*-SK Clones

**Figure 2:**
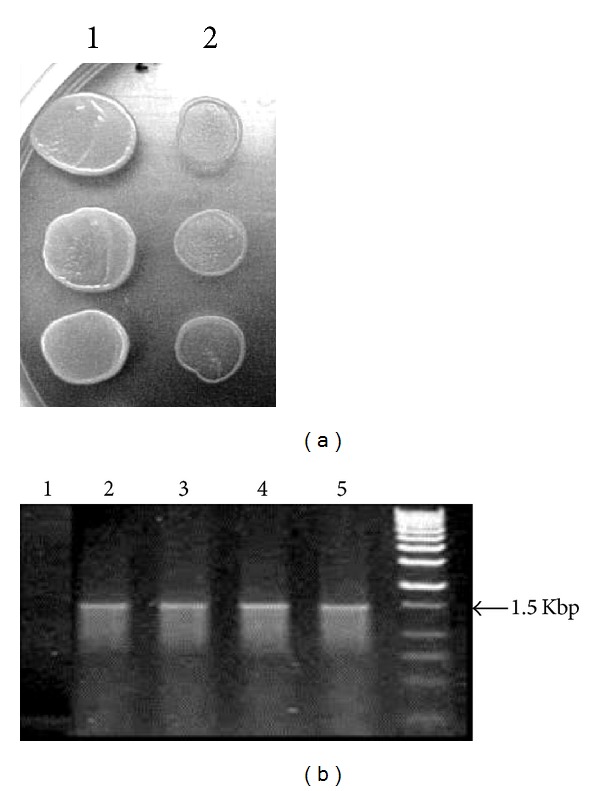
Screening of *S. cerevisiae *transformants. (a) *S. cerevisiae *transformed with *pB2ZB2*-SK expression vector were selected on YPD-Zeocin (200 *μ*g/mL) medium. Transformants with *pB2ZB2*-SK vector (Lane 1), untransformed *S. cerevisiae *(Lane 2). (b) Colony PCR (P3 and P4 primers) of *Saccharomyces* transformants selected on YPD Zeocin medium. Untransformed *S. cerevisiae *(Lane 1), transformants with 1.5 Kbp *skc* expression cassette fragments (Lanes 2, 3, 4, and 5) and 0.25–12.0 Kbp DNA ladder (Lane 6).

*NarI* linearized *pB2ZB2*-SK recombinant vector was used to transform *S. cerevisiae*. *Saccharomyces* transformants integrated with the constitutive SK expression cassette were selected on YPD agar plates containing Zeocin for 3-4 days at 30°C (Figure 2(a)). The presence of 1.5 Kbp P_*GAP*_-*skc*-_*AOX*_TT insert in *Saccharomyces* transformants was confirmed by colony PCR (Figure 2(b)). 85 transformants selected with Zeocin were analyzed for SK expression by LNA assay. 84 transformants had SK expression, and the clone (*S.cerv*-SK) with 1050 IU/mL is chosen for further experiments. Southern hybridization analysis of the *S.cerv*-SK probed with P_*GAP*_-*skc*-_*AOX*_TT DNA fragment generated a 1.63 Kbp product (Figure 4).

### 3.3. Growth and Expression Profile

**Figure 3:**
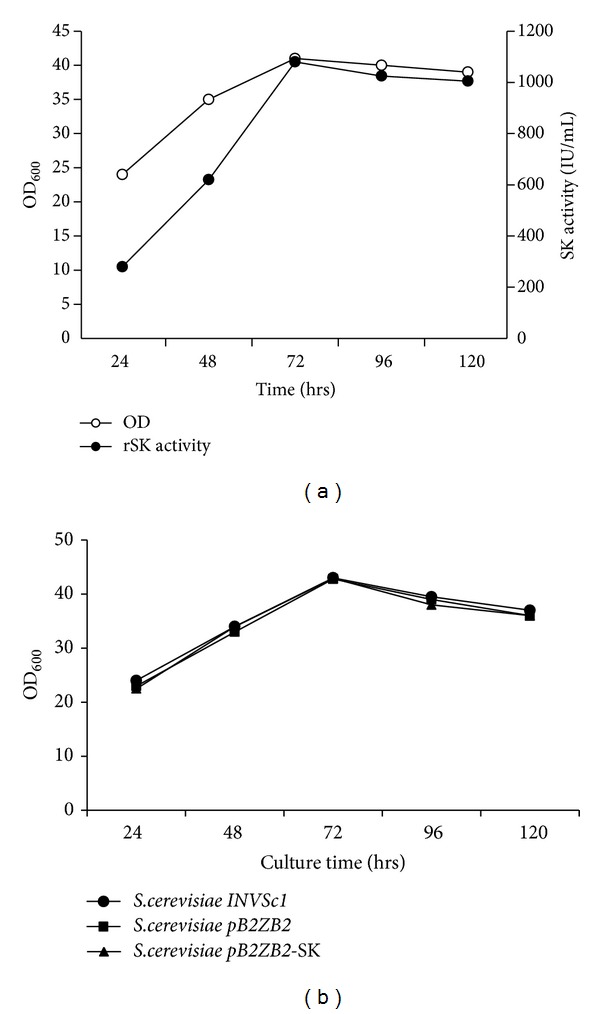
(a) Plasminogen activation assay of rSK. Growth profile and outline of SK expression of *S. cerevisiae *clone. Cells were grown in YPD medium for 5 days. Aliquots of cells were examined at different time intervals for growth and SK activity in the cell lysis supernatant by chromogenic assay using S-2251 substrate. (b) Expression of rSK has no toxic effect on the growth rate of the *S. cerevisiae *host. *S. cerevisiae *INVSc1 cells were transformed with parent plasmids and recombinant expression vector *pB2ZB2*-SK. Cells were cultured in YPD broth and aliquots were withdrawn at indicated time points up to 120 hrs, OD600 recorded and plotted. The above data is a mean of three to six independent experiments.

A comparative growth/expression analysis was performed between normal and baffled-flask cultures. Quantification of SK activity by plasminogen activation assay using cell lysates indicated an expression level of 1050 IU/mL in a normal flask and 1300 IU/mL for a baffled flask (Figure 3(a)). All the media optimization experiments were conducted with baffle-flask design.

**Figure 4:**
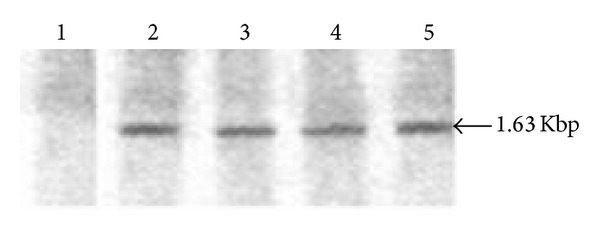
Southern blot analyses of *S. cerevisiae-*SK clone. Genomic 635 DNA of the clone was restricted with *SapI*/*AgeI *enzymes, blotted, and hybridized with *α*32P-labelled 1.63 Kbp P_*GAP*_-*skc*-_*AOX*_TT cassette fragment. *S. cerevisiae *clone genomic DNA extracted at 24 (Lane 2), 72 (Lane 3), 96 (Lane 4), and 120 hrs (Lane 5) and untransformed *S. cerevisiae* genomic DNA (Lane 1). 1.63 Kbp P_*GAP*_-*skc*-_*AOX*_TT cassette fragment is specified.

Stability and integration of *skc* gene in the *Saccharomyces* clone expressing SK were analyzed by southern blot analysis. 1.63 Kbp band is consistently seen from 24 to 120 hrs indicating stability of the continuously cultured clone (Figure 4).

The toxicity of continuous expression of SK on the host cells harboring the plasmids *pB2ZB2*-SK and *pB2ZB2* and parent untransformed cells was investigated by comparing the growth rate. No significant difference in growth rate of *S. cerv*-SK clone, compared with the controls, was observed. (Figure 3(b)). The above data indicates a correlation between the time of onset of expression of SK with an increase in the growth rate, suggesting that the expression of SK is not toxic to the host. Qualitative and quantitative analyses of rSK in cell lysis supernatant were performed by caseinolytic assay and plasminogen activation assay (S-2251 substrate) at different time intervals of growth. The results indicate that expression of rSK is observed from day 1 and is maintained till the end of the experiment.

### 3.4. Carbon and Nitrogen Sources Screening by Plackett-Burman Design

Plackett-Burman statistical design was used to test various carbon (dextrose, sucrose, fructose, maltose, galactose, lactose and glycerol) and nitrogen sources' (yeast extract, tryptone, peptone, casamino acids, beef extract corn steep liquor, and polypeptone) effect on SK production.

Pareto charts (Figures 6 and 7) were used to show the effect of various carbon and nitrogen sources on SK production. It is evident from Figure 6 that dextrose has a significant effect on SK production. The SK production in a normal flask was 1050 IU/mL and 1300 IU/mL in a baffled flask resulting in that dextrose is the most significant carbon source for SK production in a baffled flask. On the other hand glycerol had, least effect on SK production with yields 310 and 440 IU/mL in normal and baffled flasks, respectively. Further, the influence of different nitrogen sources on SK production has been shown in Figure 7. Yeast extract was most influential with 1410 and 1100 IU/mL of SK production in baffled and normal flasks, respectively. From Figures 6 and 7, it is clearly evident that the shape of the flask has a significant effect on the amount of SK produced in confirmative lines with the work done by Villatte et al. [29]. Results indicate that PB design is a powerful technique to identify carbon and nitrogen sources for better SK production. Further, CCD and RSM were used to identify the exact optimal values of the individual parameter.

### 3.5. Optimization of SK Production Medium Conditions by Design of Experiments and RSM

Experiments 16, 15, 14 and 2 (supplementary Table S4) resulted in a maximum production of SK with 2350, 2150, 2100, and 2100 IU/mL, respectively, among all combinations. Four critical independent variables, yeast extract, dextrose, pH, and temperature, were chosen to optimize the production of SK by *S. cerevisiae.* Experiments were performed according to the CCD experimental design given in supplementary Table S5 in order to search for the optimum combination of components of the medium.

From supplementary Table S5 (model summary), coefficient of determination (*R* ^2^), 0.816, indicates fitness of model which means that the statistical model can explain 81.6% of variability in the response for SK production. The *R* ^2^ value should always be between 0 and 1 and the closer the *R* ^2^ value to 1, the stronger the model to predict the response. The adjusted *R* ^2^ value is another one (supplementary Table S5) which corrects the *R* ^2^ value for the sample size and for the number of terms in the model. Here, the adjusted *R* ^2^ value is 0.645, indicating better fitness of the model used for optimization by RSM [38, 39]. The value of adjusted *R* ^2^ is smaller than *R* ^2^ if the model has many terms and the sample size is small. The values of adjusted *R* ^2^, 0.645; *R* ^2^, 0.816; and the coefficient of variation, CV = 11.13%, indicate that the model can predict precise values from the experiments carried out for SK production.

Multiple regression analysis was carried out on CCD experimental data and a second-order full polynomial equation was fitted. Equation (5) gives the empirical relationship between SK production (*Y*) and four test variables in coded units:

(5) Y=1945+89.792∗x1+87.708∗x2+53.958∗x3 −35.208∗x4−9.740∗x1x1−69.063∗x1x2 +62.813∗x1x3−47.188∗x1x4−47.240∗x2x2 +65.938∗x2x3+43.438∗x2x4−15.990∗x3x3 +150.313∗x3x4−59.740∗x4x4,

where *Y* is SK production in IU/mL, *Y* is the response of (5), and *x*_1_, *x*_2_, *x*_3_, and *x*_4_ are the coded values of the test variables: yeast extract, 2.6% (*x*_1_); dextrose, 2.7% (*x*_2_); pH, 7.1 (*x*_3_); temperature, 30°C (*x*_4_).

Results of analysis of variance (ANOVA) carried out on a second-order response surface model are given in supplementary Tables S5 and S6. Student's *t*-test and *p* values were used to estimate each coefficient (supplementary Tables S5 and S6. Student's *t* test and *p* values were used to estimate each coTables S5 and S6). Larger *t*-value and smaller *p* value are more significant to determine coefficients which means that linear effects of yeast extract (*p* < 0.006) and dextrose (*p* < 0.007) and interactive effect of pH and temperature (*p* < 0.0001) are significant in SK production.

Contour plots are the graphical representation of the model generated and were drawn to illustrate effects of independent variables and combined effects of each independent variable upon response variable. Contour plots, Figures 8(a)–8(f), help in predicting the SK production for different levels of production medium conditions. Infinite combinations of two test variables, while others held at 0 level, are indicated in contour plots.

Figures 8(a)–8(c) show the trend of SK production in IU/mL with the variation in yeast extract with respect to dextrose, pH, and temperature, and the maximum predicted SK production of 2000, 2400, and 2000 IU/mL, respectively, was observed. Figures 8(d) and 8(e) show the trend of SK production in IU/mL with the variation in dextrose with respect to pH and temperature, while a maximum SK production was predicted as of 2300 and 1900, respectively. Similarly, Figure 8(f) shows that a maximum of 2300 IU/mL SK production was predicted with the variation in pH and temperature.

Regression equation was solved by a numerical method [39, 40] and the optimal values of the four test variables in coded units are *x*_1_ = −0.72, *x*_2_ = 0.491, *x*_3_ = 7.2, and *x*_4_ = −0.681. The predicted value of *Y* (SK production, IU/mL) at these values of *x*'s was 2461.13 IU/mL. The real values of the four test variables were obtained by substituting the respective coded values in (2) and found to be yeast extract (*x*_1_), 3.215%; dextrose (*x*_2_), 2.952%; pH, 7.42 (*x*_3_), and temperature, 32.45 (*x*_4_). Further, optimized SK production medium conditions were validated in 250 mL EM flask containing 100 mL of production medium. Experiments were conducted in triplicates to check the reproducibility of SK production using optimized conditions. The SK production of 2352.07 IU/mL (average of three experiments) was obtained at optimal conditions. This shows an increase of almost 110% than before optimizing experimental conditions. The experimental value of the SK production was almost equal if we consider 95% of the confidence limits for the prediction of *Y* value at optimized conditions with the shake-flask results.

### 3.6. Streptokinase Expressed in *S. cerevisiae* Is Functionally Active

Various qualitative and quantitative assays have been developed for the detection of SK activity; casein plate assay [32], fibrin plate assay [33], chromogenic assay [19], *in vitro* clot lysis assay [31], and a combination of these assays [34] were being proposed to accurately determine the SK activity. Lyticase produced by *Arthrobacter luteus* consists of *β*-1, 3-glucanase that attacks the *β*-1, 3-glucans in a random endolytic approach releasing oligosaccharides in a pH-dependent mode [17].

Lyticase-assisted yeast estrogen screening assay was performed to evaluate the environmental toxicity of certain chemicals [18]. There are many methods available for measuring the activity of plasminogen activators: changes in clot physical structure or retainment of trapped bubbles, fibrin opacity measurement by lysis zones on plates, and changes in OD in microtiter plate formats. The common endpoint in these methods is the time to half lysis. Alternative methods use radiochemical detection or utilize modified fibrinogens with chromogenic detection. Methods using chromogenic substrates have been developed where fibrin is removed before OD measurement or involving fibrin (ogen) chemically immobilized to microtiter plates or in the presence of opaque fibrin [34].

The mechanism of quantification of SK is the conversion of inactive plasminogen to active plasmin form. Plasmin acts on the chromogenic plasmin substrate D-Val-Leu-Lys-*p*-nitroanilide and liberates *p*-nitroaniline upon cleavage [19]. Cell lysis supernatant containing SK obtained by lyticase treatment was analyzed for plasminogen activation capability by S-2251 chromogenic assay. Spectrophotometric calibration of cell density at OD_600_ was determined by direct counting in a hemocytometer or by serial titration on YPD Zeocin agar plates for viable colonies.

**Figure 5:**
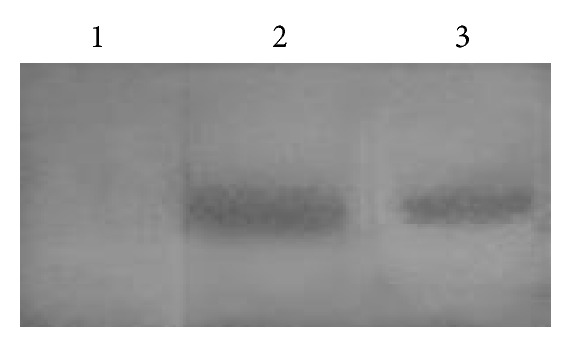
Fibrin agarose indicator gel. Untransformed *S. cerevisiae *cell lysate (Lane 1), commercial SK (lane 2), and rSK expressed in *S. cerevisiae *SK clone (Lane 3).

**Figure 6:**
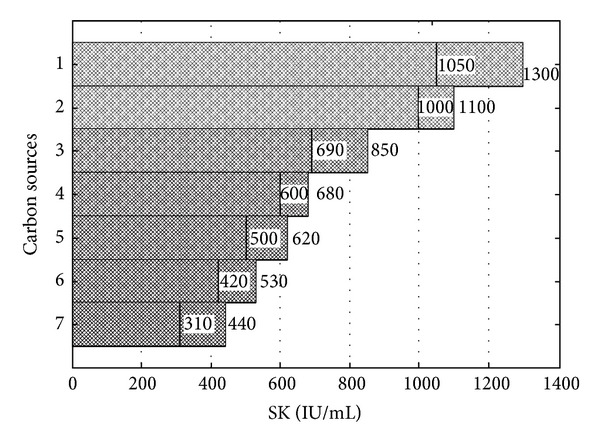
Pareto chart for the estimation of influence of different carbon sources on SK production (IU/mL) by *P. pastoris*: (1) dextrose, (2) galactose, (3) maltose, (4) sucrose, (5) lactose, (6) fructose, and (7) glycerol.

**Figure 7:**
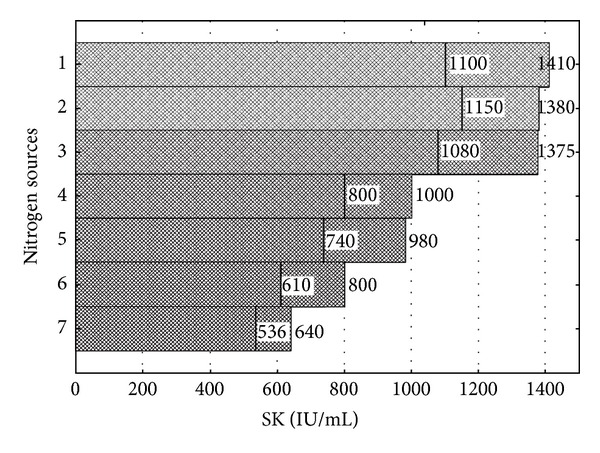
Pareto chart for the estimation of the influence of different nitrogen sources on SK production (IU/mL) by *P. pastoris*: (1) peptone, (2) yeast extract, (3) polypeptone, (4) beef extract, (5) tryptone, (6) casamino acids, and (7) corn steep liquor.

**Figure 8:**
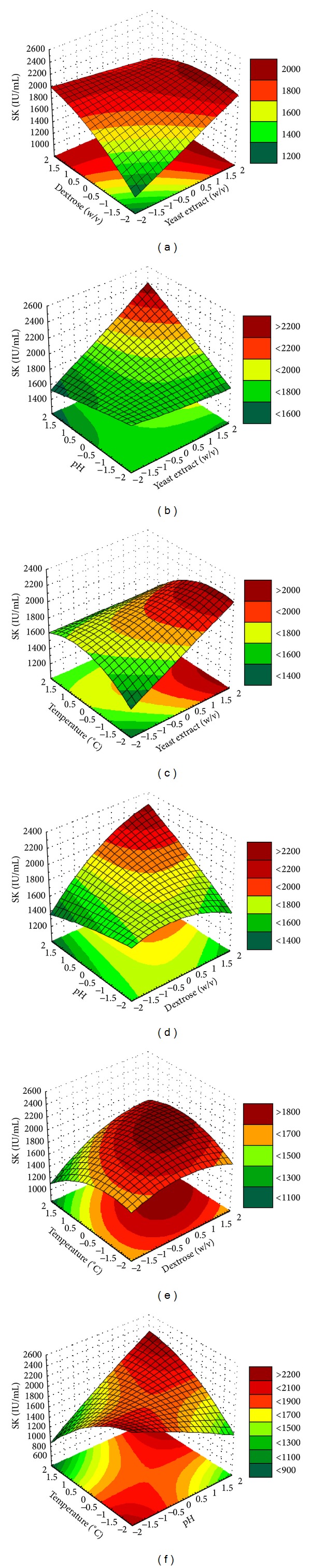
((a)–(f)) 3D surface and contour plots of SK production by *Pichia pastoris *(IU/mL): the effect of two variables while the other two are held at 0 level.

The biologically active nature of rSK expressed in *S. cerevisiae* was further analyzed by a fibrin agarose indicator clearing assay after resolving the crude protein by SDS-PAGE (Figure 5). SK resolved in PAGE activates the plasminogen, in non-enzymatic mode, in the agar gel to plasmin. Plasmin dissolves the fibrin clot formed in the agar gel evident by clear bands indicating the fibrinolytic activity. 47 kDa form of constitutively expressing SK is functional. *In vitro* clot lysis assay [34] was also used to confirm the biologically active nature of the rSK.

### 3.7. Stability of SK

Stability studies using cell lysate of *S. cerevisiae* expressing rSK at −20°C, 4°C, 25°C, 37°C, and 42°C indicated the presence of plasminogen activation efficiency at −20°C, 0°C, and 4°C (Table 1). Similar results were obtained from rSK expressed in *P. pastoris* and with SK purified from *E. coli *that was used as control.

The level of SK activity achieved by constitutive intracellular expression in *P. pastoris* is 2352 IU/mL compared to that of *Schizosaccharomyces pombe *(2450 IU/mL) [9] and* P. pastoris *(3200 IU/mL and 2089 IU/mL) [7, 14]. The optimization of nutrients at shake flask level is to be extrapolated to bioreactor level to obtain large-scale production of rSK. Continuous constitutive bioreactor culture is being investigated to achieve rSK expression at industrial scale.

## 4. Conclusion 

In conclusion, we have accomplished constitutively expressing rSK intracellularly in *S. cerevisiae *using *P. pastoris* expression backbone. The expressed SK protein (2352 IU/mL) on fibrin-plate zymography and chromogenic assay exhibited plasminogen activation property. Continuous cultivation bioreactor and purification strategies are being devised to obtain a protein of homogeneity.

## Supplementary Material

Tables S1 to S6 are statistical information used and generated while optimizing process conditions for better SK production by *Saccharomyces cerevisiae*. Table S1 and S2 are the design matrix for screening carbon and organic nitrogen sources by using Placket-Burmun design. Experiments were conducted according to the design matrix and results are used in subsequent optimization studies. Table S3 is the range and levels of four variables considered for optimization. Table S4 is the design matrix generated according to central composite design for response surface methodology. Table S5 and S6 are ANOVA of quadratic equation and responses multiple linear regression analysis respectively.

## Figures and Tables

**Table 1:** Stability of intracellularly expressed SK in *S. cerevisiae* cell lysates.

Storage temperature^a^	% Functional activity^b^ in Cell lysis supernatant
−20°C	100
0°C	94
4°C	86
25°C	9
37°C	—
42°C	—

^a^Cell lysis supernatant of Saccharomyces clone expressing SK was stored at above temperatures for duration of 96 hrs.

^b^Functional activity was assayed by *in vitro* clot lysis method and is a mean of three assays.

## Abbreviations

SK: Streptokinase
*GAP*: Glyceraldehyde 3-phosphate dehydrogenase
*AOX*: Alcohol oxidase
HRP: Horse radish peroxidase
*HIS4*: Histidinol dehydrogenase
RSM: Response surface methodology.
