# Supplementary material for: Constitutive Optimized Production of Streptokinase in Saccharomyces cerevisiae Utilizing Glyceraldehyde 3-Phosphate Dehydrogenase Promoter of Pichia pastoris
Source: Biomed Res Int. 2013 Sep 22;2013:268249. doi: 10.1155/2013/268249 (PMC3793500; doi:10.1155/2013/268249)
Supplement: Supplementary file 1 — Tables S1 to S6 are statistical information used and generated while optimizing process conditions for better SK production by Saccharomyces cerevisiae. Table S1 and S2 are the design matrix for screening carbon and organic nitrogen sources by using Placket-Burmun design. Experiments were conducted according to the design matrix and results are used in subsequent optimization studies. Table S3 is the range and levels of four variables considered for optimization. Table S4 is the design matrix generated according to central composite design for response surface methodology. Table S5 and S6 are ANOVA of quadratic equation and responses multiple linear regression analysis respectively. [file 268249.f1.pdf]

Table S1: Two level Plackett-Burman design table for the screening of carbon sources. +1 is for higher concentration and -1 for lower concentration.

Variable codes	**A**	**B**	**C**	**D**	**E**	**F**	**G**
Carbon sources	**Dextrose**	**Sucrose**	**Fructose**	**Maltose**	**Galactose**	**Lactose**	**Glycerol**
1	+1	+1	-1	+1	-1	-1	-1
2	-1	+1	-1	-1	+1	-1	+1
3	-1	-1	+1	+1	-1	-1	+1
4	-1	+1	+1	-1	-1	+1	-1
5	+1	+1	+1	+1	+1	+1	+1
6	+1	-1	+1	-1	+1	-1	-1
7	+1	-1	-1	-1	-1	+1	+1
8	-1	-1	-1	+1	+1	+1	-1

Table S2: Two level Plackett-Burman design table for the screening of organic nitrogen sources. +1 is for higher concentration and -1 for lower concentration.

Variable codes	**A**	**B**	**C**	**D**	**E**	**F**	**G**
**Organic** ***Nitrogen sources***	**Peptone**	**Yeast extract**	**Tryptone**	**Casamino acids**	**Beef extract**	**Corn steep liquor**	**Polypeptone**
1	+1	+1	-1	+1	-1	-1	-1
2	-1	+1	-1	-1	+1	-1	+1
3	-1	-1	+1	+1	-1	-1	+1

4	-1	+1	+1	-1	-1	+1	-1
5	+1	+1	+1	+1	+1	+1	+1
6	+1	-1	+1	-1	+1	-1	-1
7	+1	-1	-1	-1	-1	+1	+1
8	-1	-1	-1	+1	+1	+1	-1

Table S3: Range and Levels of the Variables in Coded Units for RSM Studies

**Variables**	**-2**	**-1**	**0**	**1**	**2**	**ΔX**
Yeast Extract, x1, % (w/v)	1.6	2.1	2.6	3.1	3.6	0.5
Dextrose, x3, % (w/v)	1.7	2.2	2.7	3.2	3.7	0.5
pH, x4	6.1	6.5	7.1	7.5	7.9	0.4
Temperature, ^{o}C	26	28	30	32	34	2

*ΔX step increment in the input variable values

Table S4: Design of Experiments by Central Composite Design (CCD) for RSM Studies

**Run number**	**x1**	**x2**	**x3**	**x4**	**Coefficients assessed by**	**SK production Measured (IU/ml)**	**SK production Predicted (IU/ml)**
1	-1	-1	-1	-1	Fractional factorial 2^4 design (16 expts)	1750	2249.79
2	1	-1	-1	-1		2100	2071.25
3	-1	1	-1	-1		1775	2068.33
4	1	1	-1	-1		1850	2166.04

5	-1	-1	1	-1		1320	1799.58
6	1	-1	1	-1		1950	1872.29
7	-1	1	1	-1		1850	1881.87
8	1	1	1	-1		2000	2230.83
9	-1	-1	-1	1		1450	1838.33
10	1	-1	-1	1		1500	1848.54
11	-1	1	-1	1		1750	1830.62
12	1	1	-1	1		1600	2117.08
13	-1	-1	1	1		1750	1989.37
14	1	-1	1	1		2100	2250.83
15	-1	1	1	1		2150	2245.41
16	1	1	1	1		2350	2783.12
17	-2	0	0	0		1750	1804.37
18	2	0	0	0		2000	2163.54
19	0	-2	0	0		1550	1958.54
20	0	2	0	0		1900	2309.37
21	0	0	-2	0		1950	1901.04
22	0	0	2	0		1750	2116.87
23	0	0	0	-2		1900	2113.54
24	0	0	0	2	8 star points	1450	2254.37
25	0	0	0	0		1950	1945.00
26	0	0	0	0		1850	1945.00
27	0	0	0	0	6 central points	2020	1945.00
28	0	0	0	0		1950	1945.00

29	0	0	0	0		1900	1945.00
30	0	0	0	0		2000	1945.00

Table S5: Model Summary and Analysis of Variance (ANOVA) for the Quadratic Model

Model estimates	Sum of Squares	Degrees of freedom	Mean Square	F value	Sig.
Regression	1254548.750	14	89610.625	4.764	0.002
Residual	282135.417	15	18809.028		
Total	1536684.167	29			

R, 0.904; $\mathbf{R^2}$, 0.816; **Adjusted** $\mathbf{R^2}$**,** 0.645; **Std. Error of the Estimate,** 137.146; **CV**, 11.13%

Table S6: Model Coefficients Estimated By Multiple Linear Regressions (Significance of Regression Coefficients)

Variables	Beta	Standard Error	t	p-value (Sig. at <0.05)
(Constant)	1945.000	55.990	34.739	0.0002
x1	89.792	27.995	3.207	0.006
x2	87.708	27.995	3.133	0.007
x3	53.958	27.995	1.927	0.073
x4	-35.208	27.995	-1.258	0.228
x1x1	-9.740	26.187	-0.372	0.715
x1x2	-69.063	34.287	-2.014	0.062
x1x3	62.813	34.287	1.832	0.087

x1x4	-47.188	34.287	-1.376	0.189
x2x2	-47.240	26.187	-1.804	0.091
x2x3	65.938	34.287	1.923	0.074
x2x4	43.438	34.287	1.267	0.225
x3x3	-15.990	26.187	-0.611	0.551
x3x4	150.313	34.287	4.384	0.001
x4x4	-59.740	26.187	-2.281	0.038
